# Supplementary material for: Stiff Extracellular Matrix Promotes Invasive Behaviors of Trophoblast Cells
Source: Bioengineering (Basel). 2023 Mar 21;10(3):384. doi: 10.3390/bioengineering10030384 (PMC10045595; doi:10.3390/bioengineering10030384)
Supplement: Supplementary file 1 [file bioengineering-10-00384-s001.zip › bioengineering-2215199-supplementary.pdf]

Supplementary Materials

Stiff Extracellular Matrix Promotes Invasive Behaviors of Trophoblast Cells

Jialing Cao [1,2], Hangyu Li [3,4], Hongyan Tang [1], Xuenan Gu [1], Yan Wang [5,*], Dongshi Guan [3,4,*], Jing Du [1,*] and Yubo Fan [1,*]

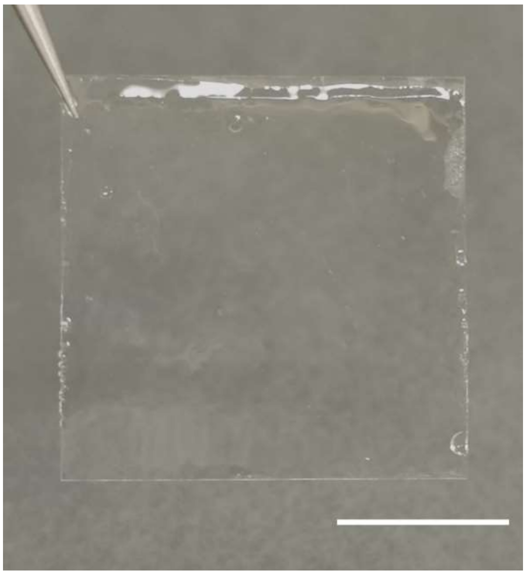

Figure S1. Fabricated sample of polyacrylamide hydrogel with stiffness gradient. Scale bar = 10 mm.

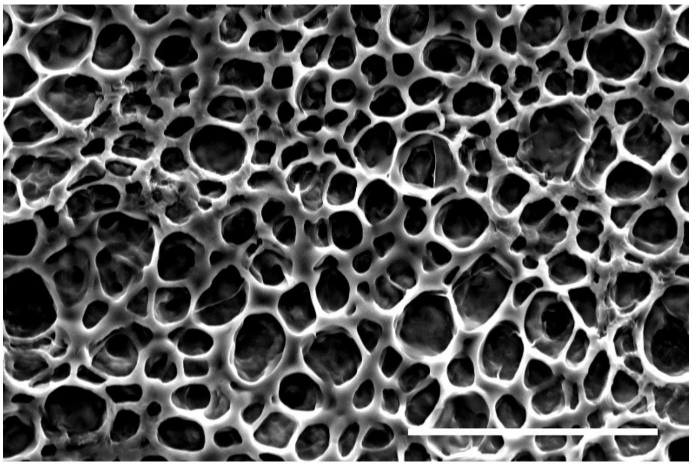

Figure S2. SEM analysis of PA gel surface. Scale bar = 30μm.

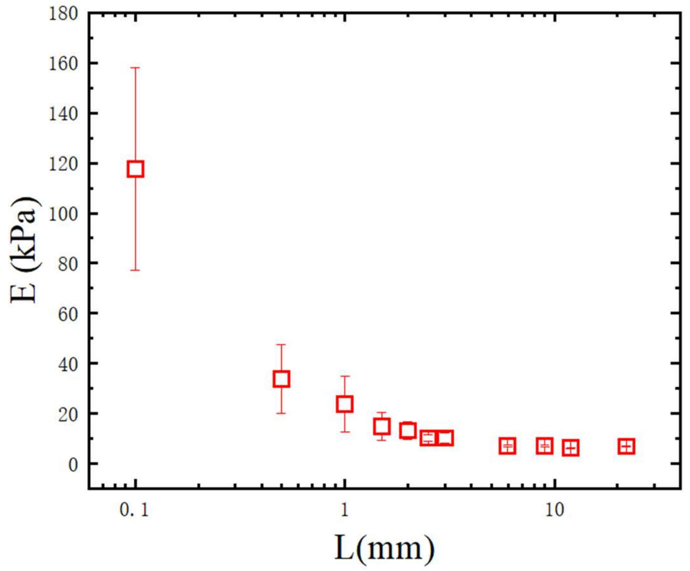

Figure S3. Elastic modulus measured by AFM. Data reported as mean ± standard deviation for N = 3 independent experiments.

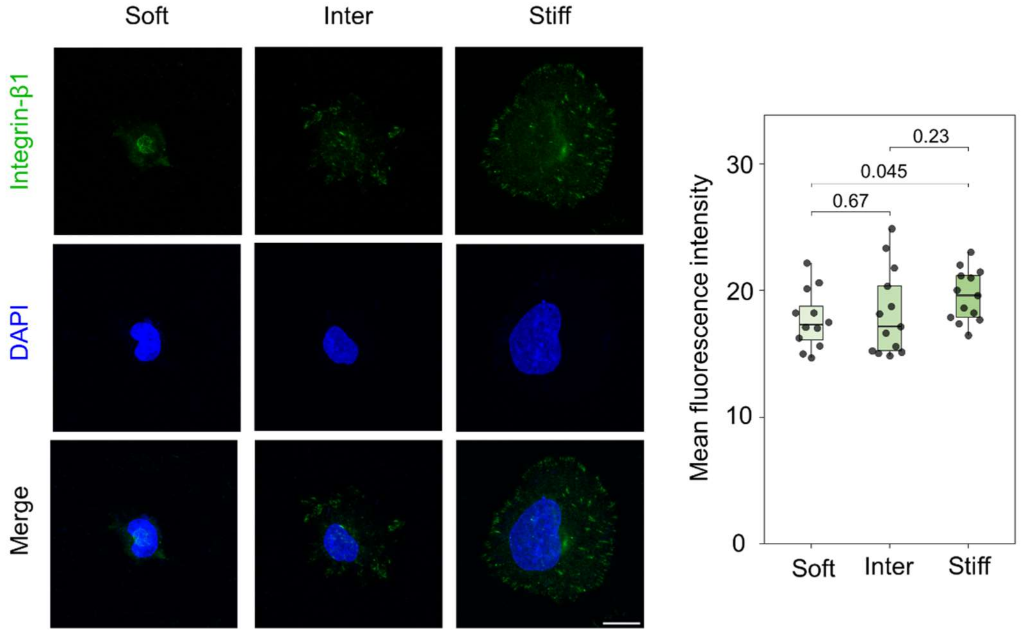

Figure S4. Left: Immunofluorescence staining of JAR cells cultured on different regions (green: integrin-β1; blue: DAPI, scale bar: 20 μm). Right: mean fluorescence intensity of integrin-β1 in JAR cells cultured on different regions. n = 12, 13, 13 for Soft, Inter and Stiff, respectively. Data reported as mean ± standard deviation.
